# Simultaneous determination of the combined and free concentrations of atorvastatin and its major metabolite *in vitro* and *in vivo* based on ultraﬁltration coupled with UPLC-MS/MS method: an application in a protein binding rate and metabolism ability study in uremic hemodialysis patients

**DOI:** 10.3389/fcvm.2024.1461181

**Published:** 2024-10-24

**Authors:** Ming-Chen Cao, Xin Huang, Bo-Hao Tang, Hai-Yan Shi, Yi Zheng, Wei Zhao

**Affiliations:** ^1^ Department of Pharmacy, The Affiliated Hospital of Qingdao University, Qingdao, China; ^2^ Department of Clinical Pharmacy, The First Affiliated Hospital of Shandong First Medical University & Shandong Provincial Qianfoshan Hospital, Jinan, China; ^3^ Shandong University of Traditional Chinese Medicine, Jinan, China; ^4^ Shandong Engineering and Technology Research Center for Pediatric Drug Development, Jinan, China; ^5^ Department of Clinical Pharmacy, School of Pharmaceutical Sciences, Cheeloo College of Medicine, Shandong University, Jinan, China

**Keywords:** atorvastatin, UPLC-MS/MS, uremia, metabolite, protein binding rate

## Abstract

**Introduction:**

A rapid, accurate, and specific ultrafiltration with ultra-performance liquid chromatographic-tandem mass spectrometry method was validated for the simultaneous determination of the protein binding rate of atorvastatin in uremic patients. Methods: The plasma samples were centrifuged at 6,000 r/min for 15 min at 37°C and the ultrafiltrate was collected. An ACQUITY UPLC® BEH C18 Column with gradient elution of water (0.1% formic acid) and acetonitrile was used for separation at a flow rate of 0.4 ml/min.

**Results:**

The calibration curves of two analytes in the serum showed excellent linearity over the concentration ranges of 0.05-20.00 ng/ml for atorvastatin, and 0.05-20.00 ng/ml for orthohydroxy atorvastatin, respectively. This method was validated according to standard US food and drug administration and European medicines agency guidelines in terms of selectivity, linearity, detection limits, matrix effects, accuracy, precision, recovery, and stability. This assay can be easily implemented in clinical practice to determine the free and combined concentrations of atorvastatin in the plasma of uremic patients. The final result showed that the average plasma protein binding rate in uremic patients was 86.58 ± 2.04%, relative standard deviation (RSD) (%) = 1.98, while the plasma protein binding rate in patients with normal renal function was 97.62 ± 1.96%, RSD (%) = 2.04. There was a significant difference in the protein binding rate in different types of plasma (*P* < 0.05), and the protein binding rate decreased with increasing creatinine until it stabilized at nearly 80%. The mean metabolite/prototype ratio of atorvastatin in patients with normal renal function and in patients with uremia was 1.085 and 0.974, respectively.

**Discussion:**

The metabolic process of atorvastatin may be inhibited in uremic hemodialysis patients, but the total concentration of atorvastatin did not change significantly; due to the decrease of protein binding rate increase the drug distribution of atorvastatin in the liver or muscle tissue, which may increase the risk of certain adverse reactions. We recommend that clinicians use free drug concentration monitoring to adjust the dose of atorvastatin to ensure patient safety for uremic hemodialysis patients.

## Introduction

After oral administration, drugs are absorbed through the intestines and enter the bloodstream. Some drugs form drug–plasma protein complexes, while others exist in a free form. Only the free form of a drug can exert pharmacological effects and participate in the body's metabolism and elimination processes. The binding of drugs to plasma proteins in the human body is in a dynamic equilibrium. The protein binding rate of drugs is generally constant under stable physiological conditions. However, under certain special or severe pathophysiological conditions, especially for drugs with high protein binding rates (>80%), the free drug concentrations may fluctuate dramatically. An increase in the free drug concentration may further expand the tissue distribution range of the drug, prolong its half-life, and increase the risk of adverse effects. In such cases, even though the total drug concentration remains within the effective therapeutic range, patients may exhibit significant individual pharmacological differences, leading to variations in the strength of pharmacological effects or the degree of adverse reactions (1). Therefore, the concentration of free-form drugs serves as a direct basis for evaluating drug safety and pharmacology. Guiding and adjusting individualized drug doses based on protein binding rates and free drug concentrations is more scientific and accurate than using total blood drug concentrations. Further research on the differences and regularities in protein binding rates and free drug concentrations in special populations is of great importance for ensuring the safety and efficacy of clinical drug use.

Chronic kidney disease is a common chronic illness in clinical practice. The progression of renal function decline in patients is usually irreversible, eventually leading to complete renal failure and the development of end-stage renal disease, known as uremia. In uremia, gastrointestinal dysfunction, autonomic neuropathy, changes in the internal environment, etc., can affect drug–protein binding rates, drug transporter activity, and enzyme metabolism, and may hinder the excretion of certain drugs, leading to increased bioavailability and decreased metabolism and excretion, affecting drug metabolism kinetics (2). In patients with uremia, the accumulation of uremic toxins in the body competes with drugs for binding sites on albumin; the uremic toxins such as aromatic amino acids, peptides, or metabolites accumulate in the body and bind to albumin in the serum to form most of the large and medium molecules that are almost impossible to remove by blood purification methods (3). This alters the surface conformation of binding proteins or causes albumin denaturation, resulting in decreased protein affinity that may alter the protein binding rates of certain drugs (2, 4). In addition, changes in body fluid pH and hypoalbuminemia during uremia also have varying degrees of impact on drug–protein binding rates (2).

Atorvastatin is a potent synthetic inhibitor of 3-hydroxy-3-methylglutaryl (HMG)-CoA reductase, the rate-limiting enzyme in cholesterol biosynthesis, and has been widely used in many countries for the treatment of hyperlipidemia because of its high efficacy and safety (5). Recent studies have found that statins play an important role in the prevention and treatment of kidney disease and have certain renal protective effects (6), thus representing a large proportion of the hypolipidemic drugs used in uremic hemodialysis patients. Atorvastatin extensively binds to plasma proteins (95%–98%) and is not effectively eliminated by conventional hemodialysis. There may be a risk of drug accumulation in uremic patients, and the protein binding rate of the drug may change, increasing the risk of medication. The incidence of rhabdomyolysis in severe adverse reactions to atorvastatin has been reported to increase with increasing blood drug concentration (7). The Lipitor® (atorvastatin calcium) specification states that kidney disease does not have any effect on atorvastatin plasma concentrations and lipid-lowering effects. Renal dysfunction does not require an adjustment of dose, but whether uremic hemodialysis patients need a dose adjustment is not yet clear.

In this study, a rapid and precise ultraﬁltration and ultra-performance liquid chromatographic-tandem mass spectrometry (UPLC-MS/MS) method was established and was used to study the changes in the protein binding rate of atorvastatin in uremic hemodialysis patients *in vitro*. This will provide a reference for the safe and effective use of medications in dialysis patients with uremia, ensuring the safety of drug administration for this special group.

## Experimental

### Chemicals and materials

The standards of atorvastatin (Lot number 132320-201405) were purchased from the National Institute for Food and Drug Control (Beijing, China). The standards of ortho-hydroxy atorvastatin (Lot number 241420) were purchased from TLC PharmaChem., Inc. (Canada). The reference standard of chlorzoxazone (internal standard, IS; Lot number 133874-201508) was purchased from the National Institute for Food and Drug Control (Beijing, China). High performance liquid chromatography-grade methanol, acetonitrile, and methyl tert-butyl ether (MTBE) were obtained from Sigma-Aldrich (St. Louis, MO, USA). Formic acid and ammonium acetate (analytical reagent) were obtained from the Beijing Chemical Factory (Beijing, China). The water used in the laboratory was ultra-pure water. All other chemicals used were of analytical reagent grade.

Amicon Centrifree ® UFC501096 micropartition devices with a filter membrane with a 10,000 KDa molecular weight cut-off were purchased from Millipore (Bedford, MA, USA).

Plasma was obtained from drug-free volunteers with normal renal function and uremic hemodialysis patients.

### Preparation of standard solutions

Stock solutions of atorvastatin (1 mg/ml), ortho-hydroxy atorvastatin (1 mg/ml), and chlorzoxazone as the internal standard (100 µg/ml) were dissolved in methanol. The stock solutions were protected from light and kept at 4°C until used. The stock solutions were successively diluted with methanol:water (50:50, V:V) to prepare working solutions just prior to use. These solutions were spiked into drug-free human plasma samples to give ﬁnal concentrations of 0.050, 0.10, 0.20, 0.50, 1.00, 2.50, 5.00, and 20.00 ng/ml.

### Collection and preparation of plasma samples

All the patients included in the study were uremic patients undergoing regular hemodialysis three times a week, with a 2-day interval between each session. Blood samples were taken before hemodialysis to measure the atorvastatin concentrations in uremic patients. Each plasma sample (400 µl) was immediately mixed with 20 μl of methanol-water (50:50, v/v), 40 µl of the internal standard solution, and 10 μl of 0.1 M sodium acetate solution; vortexed for 30 s; and then mixed with MTBE 1 ml. After vortex mixing for 1 min, the samples were centrifuged for 15 min at 6,000 g on a Thermo Fisher Scientific Sorvall ST 16R centrifuge and 800 μl of the upper MTBE fraction was transferred into a 1.5 ml EP tube. The upper MTBE fraction was evaporated to dryness under a gentle stream of nitrogen. The residue was redissolved in 100 µl of methanol-water (50:50, v/v), vortexed for 1 min, then centrifuged at 13,800 g for 10 min. The supernatant was then sampled and 10 µl was injected into the UPLC system for analysis.

#### Instrumentation and chromatographic conditions

Analyses were performed on an MS/MS system consisting of an AB SCIEX QTRAP 6500plus MS/MS, and a UPLC system consisting of a binary pump, an autosampler, and an online degasser was used for the UPLC-MS/MS analysis (SCIEX, MA, USA). Separation was achieved using an ACQUITY UPLC® BEH C18 Column (1.7 μm, 2.1 mm × 30 mm, ID) and ACQUITY UPLC® BEH C18 VanGuardTM Pre-Column 3/PK (1.7 m, 2.1 mm × 5 mm) purchased from Waters (MA, USA). Gradient elution was performed using water (0.1% formic acid, PH 2) (A) and acetonitrile (B) at a flow rate of 0.4 ml/min. The mobile phase was filtered prior to use through a 0.22 µm Millipore filter paper (Billerica, MA, USA) and degassed ultra-sonically (Branson-Emerson USA) for 10 min. The gradient program was as follows: 0.0–0.5 min 90% A; 0.5–1.5 min, 90% A to 5% A; 1.5–2.0 min, 5% A; 2.0–2.1 min, 5% A to 90% A; 2.1–3.5 min, 90% A. The pressure of the UPLC apparatus was in the range of 9.5–19.5 MPa.

The ESI was operated in the positive ion mode. Multiple reaction monitoring (MRM) was optimized by the flow injection analysis (FIA) mode: source temperature (TEM), nebulizer (NEB), curtain (CUR), and auxiliary (AUX) gas were set at 40, 40, 20, and 40 psi, respectively. Nitrogen gases were used as the collision and curtain gases while zero air was used as the source gas. Determinations of atorvastatin and ortho-hydroxy atorvastatin were based on the internal standard method, using chlorzoxazone as the IS. Column oven temperature (40°C) and injection volume (10 µl) were maintained throughout the analysis. The MRM mode was used to qualify at m/z 559.3, m/z 575.3, and m/z 170.1, and quantify the target compounds at m/z 440.0, m/z 440.1, and m/z 114.3 for atorvastatin, ortho-hydroxy atorvastatin, and chlorzoxazone, respectively. The main working parameters of the MS were optimized as follows: ion spray voltage was at 5,500 V and source temperature was at 450°C. Nitrogen gas was used as the sheath gas (55 arbitrary units) and auxiliary gas (15 arbitrary units). The declustering potential (DP) was set at 55 V, the collision energy was optimized at 26 eV for atorvastatin, 30 eV for ortho-hydroxy atorvastatin, and 20 eV for IS. The developed method and aforementioned instruments were used for all the experiments conducted in this study and the specifications of other instruments used are mentioned where required.

### Method validation

#### Selectivity

The selectivity was defined as the absence of interference from the blank serum components at the retention times of atorvastatin, ortho-hydroxy atorvastatin, and IS using the proposed extraction procedure and UPLC/MS conditions. Six different blank serum samples (i.e., did not receive the treatment of atorvastatin) from hospitalized volunteers with normal renal function or uremic hemodialysis patients were evaluated to assess the selectivity of the method.

#### Linearity

Prepared blank plasma samples were spiked to achieve standard series samples with plasma concentrations equivalent to 0.050, 0.10, 0.20, 0.50, 1.00, 2.50, 5.00, and 20.00 ng/ml for atorvastatin and ortho-hydroxy atorvastatin. Each concentration was analyzed in duplicate by plotting the ratio of the peak areas of the analyte and internal standard against the analyte concentration. Regression analysis using the weighted (W − 1/X2) least-squares method was performed to establish the standard curve, which was validated for 3 consecutive days. The calibration curve was developed using the following criteria: (1) the mean value should be within ±15% of the theoretical value, except at the lower limit of quantification (LLOQ), where it should not deviate by more than ±20%; (2) the precision of the mean value should not exceed a 15% coefficient of variation (CV), except for LLOQ, where it should not exceed 20%.

#### Detection limits (LLOQ)

The detection limit was determined as a signal/noise ratio of at least 5. The analyte peak should be identifiable, discrete, and reproducible with a precision of 20% and an accuracy of 80%–120%.

#### Matrix effects

Matrix effects were investigated using the post-extraction spike method, which measures the ionization recovery, and was determined by the ratio of the peak area of analytes spiked after extraction to the peak area of standard solutions at the same concentration. To evaluate the relative matrix effects, calibration curves from six serum batches were constructed, and the precision [expressed by Relative Standard Deviation (RSD)] values for the slopes were calculated. The RSD should not exceed 3%–4% to confirm a method is practically free from the relative matrix effect (8).

#### Accuracy and precision

The intra-day and inter-day assay precisions were determined using the CV (%), and the accuracies were expressed as the percent difference by using the following formula:

$$\frac{\mathrm{measured}\;\mathrm{concentration}}{\mathrm{nominal}\;\mathrm{concentration}}\times 100\text{\%}$$

Intra-day assay precision and accuracy were calculated using six determinations of the three quality control (QC) (0.15, 5.00, and 15.00 ng/ml for atorvastatin and ortho-hydroxy atorvastatin) during a single analytical run. Inter-day assay precision and accuracy were calculated by analyzing the three QC (*n* = 6) on three separate days. Precision should not exceed 15% and bias should be between 85% and 115%.

#### Recovery

The recovery was determined by the analysis of serum samples at three concentrations (0.15, 5.00, and 15.00 ng/ml for atorvastatin and ortho-hydroxy atorvastatin). Each concentration level was extracted and analyzed, and the responses were compared with those of non-extracted standards corresponding to 100% recovery.

#### Stability

Stability procedures were defined to evaluate the stability of the analytes during sample collection and handling.

Short-term stability was determined by assaying the three QC samples at room temperature after thawing for 1 h.

Freeze–thaw stability was determined by assaying the three QC over three freeze–thawing cycles. The QC samples were stored at −80°C for 24 h and thawed at room temperature. When completely thawed, the samples were refrozen for 24 h. The freeze–thawing cycle was repeated twice and the analysis was conducted at the end of the third cycle. The measured concentration was then compared to the theoretical concentration.

Post-preparative stability was determined by assaying the three QC samples in an autosampler at +4°C by injecting extracts immediately after preparation and re-injecting 2 and 12 h later.

Long-term stability was determined from serum samples stored at −80°C for 30 days.

### Determination of drug concentration in ultrafiltrate

A 500 µl volume of the drug-free human plasma was transferred to the Amicon Centrifree® micropartition device and centrifuged at 6,000 g (37°C) for 15 min, and all of the original volume of the plasma was collected as an ultrafiltrate. The atorvastatin standard solutions were diluted with the ultrafiltrate to 0.05, 0.50, and 2.00 ng/ml, respectively. The drug-containing ultrafiltrate was processed and analyzed the same as the plasma samples according to the method described previously, six times in parallel determination. The atorvastatin concentration in the ultrafiltrate was calculated using the standard curve of the plasma samples and was compared with the actual concentrations.

### Plasma protein binding study of atorvastatin *in vitro*

A certain amount of atorvastatin working solutions was added to the drug-free plasma (from volunteers with normal renal function and uremic hemodialysis patients, respectively.) and drug-containing plasma with concentrations of 0.05, 2.00, and 10.00 ng/ml, respectively, was prepared. To achieve equilibrium between the drug and plasma proteins, the spiked drug-containing plasma samples were incubated at 37°C for 60 min prior. An aliquot (500 μl) of the obtained plasma was transferred to the centrifugal filter unit. Samples of 500 µl volume were transferred to the Amicon Centrifree® micropartition devices and centrifuged at 6,000 g (37°C) for 15 min, and all of the original volume of plasma was collected as an ultrafiltrate (9, 10). The ultrafiltrate volume was calculated using the weight loss method and the protein binding rate was calculated according to the formula below. Plasma samples without ultrafiltration and their respective ultrafiltrates were analyzed using the UPLC-MS/MS method. The percentage of plasma protein binding (PPB) was calculated as follows:

$$\mathrm{PPB}=[(C_{\mathrm{ultra}\text{-}\mathrm{filtrate}}\times V_{\mathrm{ultra}\text{-}\mathrm{filtrate}})/V_{\mathrm{ultra}\text{-}\mathrm{filtered}\;\mathrm{plasma}}]/C_{\mathrm{plasma}}$$

The PPB and metabolism ratio study *in vivo*.

The patients in this study were all older than 18 years. The serum creatinine (SCr) of the patients with normal renal function was 54–106 and 44–97 μmol/L for men and women, respectively. The glomerular filtration rate (GFR) of the uremic patients was < 15 ml/(min 1.73 m^2^). We excluded patients with adverse reactions to atorvastatin and organ-transplant patients. All of the patients were administered atorvastatin calcium tablets (Lipitor®) for ≥7 days orally (>5 t_1/2,_ steady-state plasma concentration). The frequency of administration was once per night (q.n.) and the dose was 20 mg. Venous blood was collected at 6 a.m. the next day and placed in a heparin anticoagulation tube. The free and combined concentrations of atorvastatin and its metabolite in the samples were determined by the UPLC-MS/MS method and the percentage of PPB and the metabolite/prototype ratio were calculated. This study was designed in accordance with legal requirements and the Declaration of Helsinki and was approved by the local research ethics committee.

## Results

### Method validation

#### Selectivity

**Figure 1:**
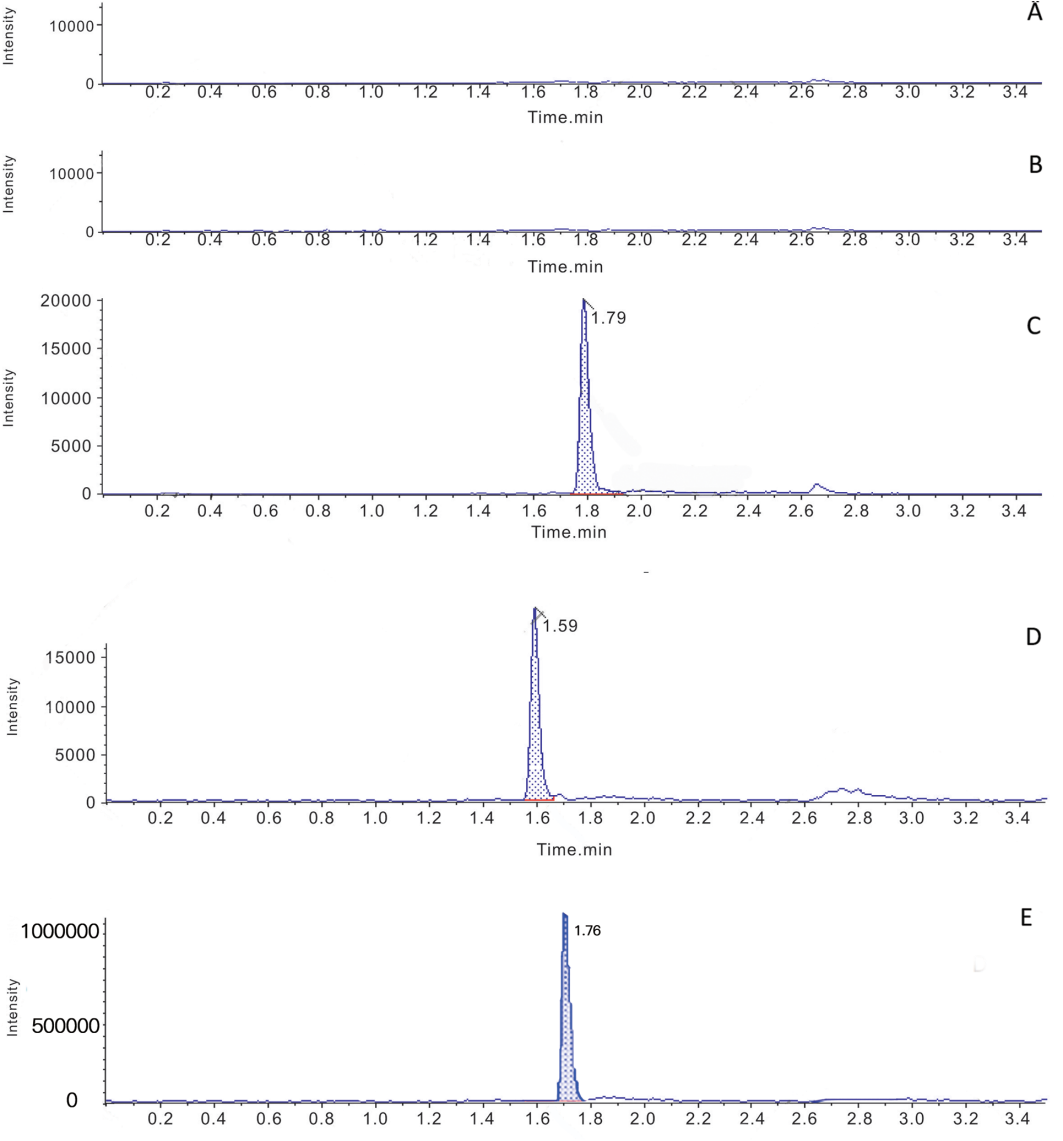
Typical multiple reaction monitoring chromatograms for atorvastatin, ortho-hydroxy atorvastatin and the IS in human plasma. **(A)** Blank plasma samples from volunteers with normal kidney function. **(B)** Blank plasma samples from uremic hemodialysis patients. **(C)** Blank plasma from uremic hemodialysis patients spiked with atorvastatin. **(D)** Blank uremia hemodialysis patients plasma spiked with ortho-hydroxy atorvastatin. **(E)** Blank plasma from uremic patients spiked with IS.

The chromatograms of the blank serum samples from the drug-free hospitalized volunteers and uremic hemodialysis patients are presented in Figure 1. No interferences were observed at the retention times of atorvastatin, ortho-hydroxy atorvastatin, and the IS.

#### Linearity

The best linear fit and least-squares residuals for the calibration curve were achieved with a 1/*x*^2^ weighting factor with linear ranges of 0.05–20.00 ng/ml for atorvastatin and 0.05–20.00 ng/ml for ortho-hydroxy atorvastatin. The linear regression equations were *y* = 0.09914*x* + 0.03348 (*r*^2 ^= 0.9980) for atorvastatin and *y* = 0.10033*x* + 0.01922 (*r*^2 ^= 0.9976) for ortho-hydroxy atorvastatin. The intercept coefficients were not significant (*p* = 1.000 for atorvastatin, *p* = 0.948 for ortho-hydroxy atorvastatin).

#### Detection limit

The lower limit of quantification was 0.05 ng/ml for atorvastatin (*S*/*N* > 5) and 0.05 ng/ml for ortho-hydroxy atorvastatin (*S*/*N* > 5).

#### Matrix effects

The three QC standard solutions (0.15, 5.00, and 15.00 ng/ml for atorvastatin and ortho-hydroxy atorvastatin) were added into three different blank serum samples in triplicate, separately. The absolute value and ratio of the peak areas of the analytes spiked after extraction to those of the standard solutions were calculated. The results showed that the matrix effects of atorvastatin and ortho-hydroxy atorvastatin on the internal standard-corrected samples were 96.23%–104.37% and 92.64%–103.59%, respectively. The RSD (%) of both were in the normal range (did not exceed 3%–4%). Therefore, the assay established in this experiment was not affected by the matrix effect.

#### Accuracy, precision, and recovery

The results of the recovery, intra-day and inter-day precision, and accuracy of atorvastatin and ortho-hydroxy atorvastatin are given in Table 1.

**Table 1:** Precision and recovery for the analysis of atorvastatin and ortho-hydroxy atorvastatin in plasma from uremic hemodialysis patients (*n* = 6).

Item	Concentration (ng/ml)	Intra-day ($\bar{x}\pm s$)	RSD (%)	Inter-day ($\bar{x}\pm s$)	RSD (%)	Recovery ($\bar{x}\pm s$)
Atorvastatin	0.15	0.143 ± 0.009	3.35	0.147 ± 0.006	4.23	84.1 ± 2.3%
	5.00	5.004 ± 0.043	1.43	5.039 ± 0.047	2.85	76.5 ± 1.1%
	15.00	14.88 ± 0.16	0.65	14.85 ± 0.21	1.66	82.0 ± 1.7%
Ortho-hydroxy Atorvastatin	0.15	0.143 ± 0.008	4.56	0.146 ± 0.005	5.22	84.4 ± 2.5%
	5.00	4.915 ± 0.080	2.08	4.963 ± 0.11	2.34	79.1 ± 1.5%
	15.00	15.07 ± 0.12	0.99	15.04 ± 0.17	1.55	82.0 ± 1.7%

### Stability

The short-term stability and three consecutive freeze–thawing cycles showed no significant degradation for atorvastatin and ortho-hydroxy atorvastatin. The result of the post-preparative stability analysis showed that atorvastatin and ortho-hydroxy atorvastatin were still stable 2 and 12 h after operation. The serum samples stored at −80°C were found to be stable for 30 days.

### Determination of drug concentration in ultrafiltrate

The RSD between the concentration calculated from the standard curve (plasma) and the actual concentration was less than 5.57%. The statistics analysis showed that there was no significant difference (*P* < 0.05). This method is suitable for the determination of the free concentration of atorvastatin in the ultrafiltrate.

### Protein binding rate experiment

The study *in vitro* (Table 2) showed that the plasma protein binding rate in the plasma of uremic patients was reduced by approximately 10% compared with that in the plasma of the patients with normal renal function. The average plasma protein binding rate in the uremic patients was 86.58 ± 2.04%, RSD (%) = 1.98, while the protein binding rate in the plasma of patients with normal renal function was 97.62 ± 1.96%, RSD (%) = 2.04. A *t*-test showed that there was no significant difference in the protein binding rate of atorvastatin at different concentrations (*P* > 0.05), but there was a significant difference in the protein binding rate in different types of plasma (*P* < 0.05).

**Table 2:** The protein binding rate of atorvastatin in the plasma from uremic patients and health volunteers at different concentrations (*n* = 6).

Concentration (ng/ml)	Healthy human plasma			Uremic patient plasma		
	1.00	5.00	10.00	1.00	5.00	10.00
Protein binding rate	98.33%	98.81%	96.56%	88.45%	86.33%	87.36%
	99.04%	96.56%	97.37%	87.83%	85.84%	86.51%
	96.27%	97.77%	95.67%	84.92%	84.49%	85.45%
	97.76%	97.83%	98.86%	86.43%	89.33%	87.84%
	95.44%	98.58%	99.05%	89.56%	84.56%	88.65%
	97.86%	98.68%	96.73%	84.56%	83.78%	86.47%
Mean	97.62%			86.58%		
RSD (%)	1.96			2.04		

### The PPB and metabolism ratio study *in vivo*

**Figure 2:**
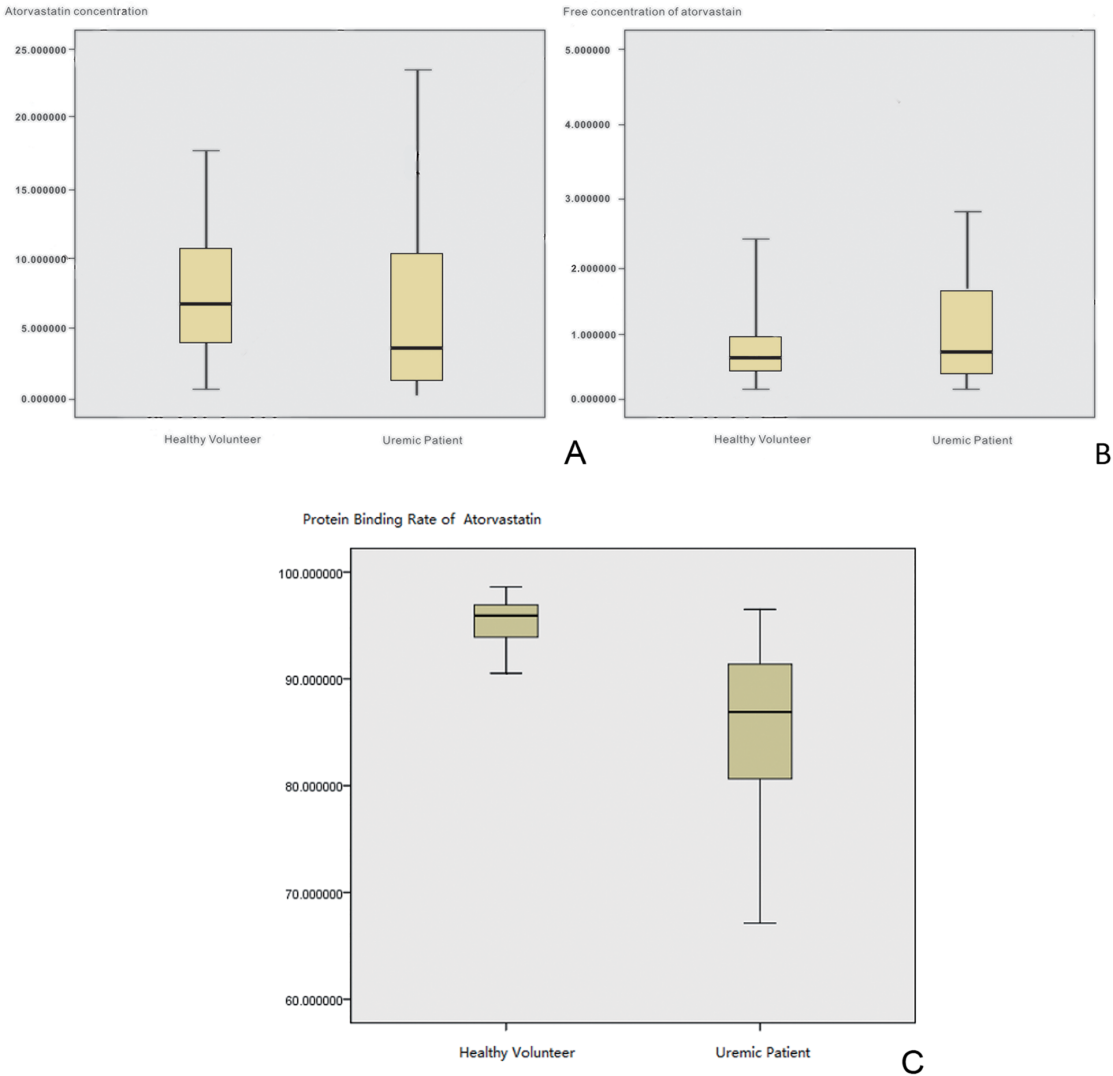
The PPB and metabolism ratio study *in vivo*. **(A)** Box-whisker plot of total plasma concentrations of atorvastatin. **(B)** Box-whisker plot of free atorvastatin concentrations. **(C)** Box-whisker plot of the protein binding rate of atorvastatin.

**Figure 3:**
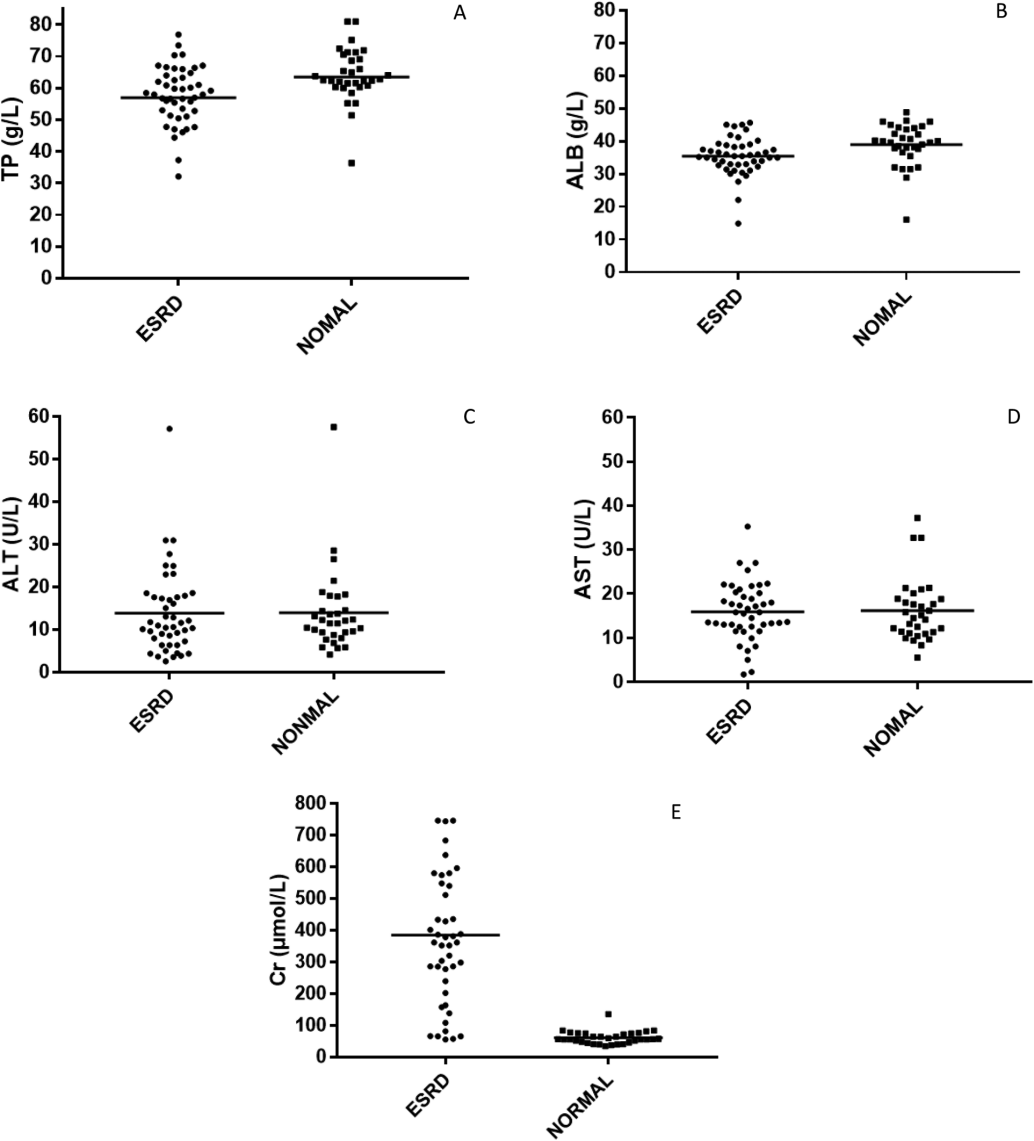
The scatter plot of the biochemical indicators. **(A)** Total protein. **(B)** Albumin. **(C)** Alanine Aminotransferase. **(D)** Aspartate Aminotransferase. **(E)** Creatinine.

**Figure 4:**
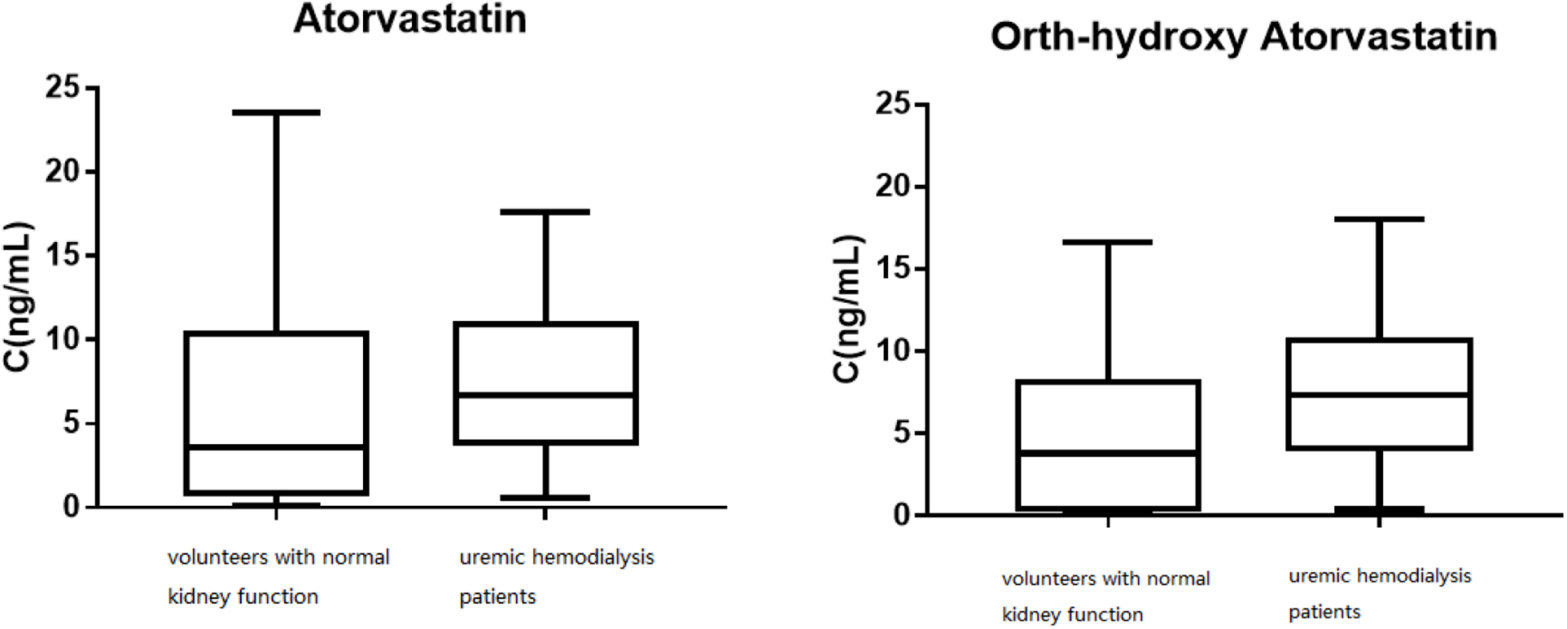
The distribution of the trough concentrations for atorvastatin and ortho-hydroxy atorvastatin.

A total of 45 uremic hemodialysis patients were included with a median age of 71 years old (range: 60–85), and 35 patients with normal kidney function were included with a median age of 67 years old (range: 50–80); both groups were administered a dose of 20 mg/day. The duration of hemodialysis treatment was evenly distributed across the age groups among the uremic patients. Statistical analysis indicated that there was no significant difference in age, gender, and BMI values between the two groups (*P* > 0.05), and the balance of baseline characteristics between the groups was good, ensuring comparability. Our study showed that the mean total plasma concentration of atorvastatin in the normal renal function group was 7.45 ± 4.68 and 5.97 ± 4.20 ng/ml for the uremic patients group (Figure 2). The *T*-test analysis showed that there was no significant difference between the two groups (*P* = 0.239 > 0.05), which means that uremia had no significant effect on the total plasma concentration of atorvastatin. However, there was a significant difference in the free concentration of atorvastatin between the two groups (*P* = 0.013 < 0.05), which was 0.38 ± 0.38 and 0.78 ± 0.94 ng/ml for the normal renal function group and the uremic patients group, respectively (Figure 3). The free concentration was significantly increased due to the decrease in the protein binding rate. The mean PPB rate of atorvastatin in the normal renal function group was 95.33% ± 2.36%, which proved that the method established in our study was trustworthy. The mean PPB rate of atorvastatin in the uremic patients group was only 85.06% ± 8.32% (Figure 4). The plasma protein of atorvastatin in the uremic patients group was significantly lower than the normal renal function group (*P* = 0.00 < 0.05). The results were consistent with the experiments *in vitro*.

Owing to the renal filtration function loss and negative nitrogen balance in uremic patients, their physiological state is often monitored using albumin and creatinine values. The serum creatinine value reflects the level of toxin accumulation in uremic patients, while albumin concentration may affect the protein binding rate of the drug.

The biochemical indicators of the subjects enrolled in this study are shown in Table 3 and Figure 3. Logistic regression analysis showed that the most significant factor related to the protein binding rate of atorvastatin was creatinine level (*P* < 0.01), and the protein binding rate decreased with increasing creatinine until it stabilized at nearly 80%. The correlation between the protein binding rate of atorvastatin and plasma albumin level was not significant in our study (*P* > 0.05).

**Table 3:** The biochemical indicators of the uremic patients and healthy volunteers.

Categories	Mean		*P*
	Healthy volunteers	Uremic patients	
AST	15.77 ± 3.21	16.02 ± 4.65	>0.05
ALT	14.55 ± 2.88	15.89 ± 3.93	>0.05
Albumin (g/L)	38.99 ± 6.43	35.37 ± 5.78	<0.05
Creatinine (μmol/L)	61.65 ± 19.82	385.89 ± 223.77	<0.01

ALT, Alanine Aminotransferase; AST, Aspartate Aminotransferase.

Figure 4 shows the distribution of the trough concentrations of atorvastatin and ortho-hydroxy atorvastatin. The average plasma concentration of ortho-hydroxy atorvastatin in the patients with normal kidney function was 7.75 ± 4.93 ng/ml and in the uremic hemodialysis patients, it was 4.97 ± 4.89 ng/ml. *T*-test analysis results showed that there was a significant difference between the two groups of plasma concentrations (*P* < 0.05). The mean metabolite/prototype ratio of atorvastatin for patients with normal renal function and those with uremia was 1.085 and 0.974, respectively, and there was a significant difference (*P* < 0.05). The metabolite/prototype ratio of the uremic patients group was lower than the normal renal function group, suggesting that the metabolic process of atorvastatin in uremic patients may be inhibited.

## Discussion

This study *in vitro* showed that the average plasma protein binding rate in uremic patients was 86.58% ± 2.04%, while the plasma protein binding rate in patients with normal renal function was 97.62% ± 1.96%. The results *in vivo* were consistent with the *in vitro* results and were 85.06% ± 8.32% and 95.33% ± 2.36%, respectively, with a significant difference (*P* < 0.05). There was a significant difference in the free concentration of atorvastatin between the two groups (*P* < 0.05), which was 0.38 ± 0.38 and 0.78 ± 0.94 ng/ml for the normal renal function group and the uremic patients group, respectively. It was evident that a 10% decrease in protein binding results in an almost 100% increase in the free drug concentration. Elevated free concentrations may be accompanied by elevated distribution to tissue concentrations, and tissue concentrations of atorvastatin are known to be abnormally high under certain specific circumstances. For example, Knuuttila et al. (11) found that measurable atorvastatin concentrations in the prostate support atorvastatin's ability to access the prostate from the circulation. Atorvastatin may accumulate in the prostate as intraprostatic concentrations are elevated compared to the plasma concentration; the median atorvastatin concentration was 212% higher in the tissue (17.6 ng/g) compared to the plasma (3.6 ng/ml). Furthermore, atorvastatin lactone concentration was 590% higher in the tissue as compared to the plasma concentration (12). This also means that atorvastatin will have a higher free concentration in the prostate of uremic patients. The mean total plasma concentration of atorvastatin in the normal renal function group was 7.45 ± 4.68, and 5.97 ± 4.20 ng/ml for the uremic patients group, which meant that uremia had no significant effect on the total plasma concentration (*P* > 0.05). However, the average plasma concentration of ortho-hydroxy atorvastatin in the normal renal function group and the uremic patients group was 7.75 ± 4.93 and 4.97 ± 4.89 ng/ml, respectively, with a mean metabolite/prototype ratio of 1.085 and 0.974, respectively, which showed that there were significantly different concentrations (*P* < 0.05). It is suggested that the metabolic process of atorvastatin may be inhibited in uremic patients. We also demonstrated that a low dose of atorvastatin was similar to medium and high doses, similar to the study by Robert L. Lins in which the pharmacokinetic parameters of atorvastatin were not significantly different between the 40 and 80 mg doses in uremic dialysis patients compared with healthy volunteers and were also proportional to the dose of the major active metabolite, o-hydroxy atorvastatin, with no accumulation and relatively low levels of the active metabolite (12).

The metabolic processes of atorvastatin were inhibited in the uremic hemodialysis patients. Atorvastatin is mainly metabolized by the CYP3A isoenzyme in the liver and can be absorbed by P-gp secretion and the H+-MCT co-transporter on the Caco-2 cell lumen side, or as a substrate for OATP1B1, OATP1A4, and OATP1B2 (13). Studies have shown that uremic toxins affect the mRNA, protein expression, and function of metabolic enzymes and drug transporters. This leads to reduced non-renal clearance of drugs in the body indicated by increased bioavailability and/or elevated plasma concentrations (3, 14, 15). Changes in the activity of renal transporters affect the pharmacokinetics of drugs that are primarily eliminated from the kidneys, and changes in liver and intestinal transporter activity affect the pharmacokinetics of non-renal elimination drugs. In addition to changes in liver metabolic enzyme activity, decreased liver drug intake or increased efflux may downregulate drug metabolism, while drugs absorbed in the intestine need to be metabolized by the gastrointestinal tract before liver metabolism and the expression of intestinal uptake transporters is upregulated. Enhanced or reduced efflux transporter activity may increase the bioavailability of certain drugs in uremic patients.

Studies have shown that renal insufficiency affects the activity of CYP450 enzymes (16–22). The total amount of CYP enzyme in mice with renal insufficiency decreased by 47% and was negatively correlated with renal clearance; the protein expression of CYP2C11, 3A1, and 3A2 was downregulated by 40%, 74%, and 65%, respectively. Furthermore, the mRNA expression levels of CYP1A2, 2C11, 2C29, 3A1, 3A2, and 3A11 were significantly downregulated (17–19). The activity and protein expression levels of CYP3A and CYP2C11 in the liver of uremic patients decreased with the downregulation of mRNA expression levels (21). Thomson et al. (22) found that the blood concentration of the CYP3A4 probe drug midazolam in hemodialysis patients increased nearly six times compared with that in people with normal renal function, suggesting that liver CYP3A4 enzyme activity is inhibited.

The liver transporter in uremic patients reduces drug intake and increases efflux, which may be one of the causes of drug metabolism downregulation; the expression and activity of uptake transporters in the intestinal tract are upregulated, and the efflux transporter activity is downregulated, affecting its transport substrate metabolism and excretion and ultimately affecting the drug concentration. Nolin et al. (23) found that the pharmacokinetic parameters of the oral administration of midazolam (CYP3A substrate) after hemodialysis in uremic patients were not significantly different from those in healthy subjects, whereas the clearance rate of oral fexofenadine (a common substrate for CYP3A, OATP, and P-gp) was reduced by 63%. Fexofenadine did not change significantly in non-hemodialysis uremic patients, while the concentration of fexofenadine in uremic patients treated with hemodialysis and peritoneal dialysis significantly increased (24), suggesting that the intestinal and liver transporters were inhibited in uremia. Furthermore, the activity of P-gp and MRP2 in the intestinal tracts of rats with renal insufficiency was reduced by 30% and 25%, respectively, and the protein expression of P-gp, MRP2, and MRP3 was decreased by more than 40%. Furthermore, the protein expression and mRNA level of the efflux transporter P-gp in the livers of renal dysfunction mice increased significantly, while the protein expression of the uptake transporter OATP2 decreased by 35% but the mRNA level did not change significantly (25–27). The expression levels of mRNA and protein in OAT1, OAT2, OAT3, OATP1, and OATP4c1 were downregulated in the kidneys, while the mRNA and protein expression levels of MRP2, MRP3, MRP4, and OATP2 and OATP3 were upregulated (28). Clinical studies have further confirmed these findings. Sakurai et al. (29) found that OAT1 mRNA expression was downregulated in live kidney sections of patients with various renal dysfunctions with varying degrees of renal insufficiency. Furthermore, OAT3 was slightly upregulated, and there was no significant difference in OAT2 and OAT4 expression, whereas the clearance rate of cefazolin (the anionic drug) was significantly correlated with the expression level of OAT3 mRNA.

In theory, when the protein binding rate of atorvastatin is reduced in uremic patients, the free drug concentration increases, the amount of the drug involved in metabolism increases, and the metabolic rate should be accelerated, but the metabolism of atorvastatin in uremic patients is inhibited. The concentration of metabolites is also significantly reduced. The presumed reasons may be as follows: (1) The CYP3A4 enzyme activity in uremic patients was inhibited, and a decrease in this activity led to a decrease in the metabolism of atorvastatin. (2) Atorvastatin was mainly transported to the liver through OATP1B3 for metabolism. The activity of OATP transporters in the liver of uremic patients was inhibited, resulting in a decrease in atorvastatin transported to liver cells via OATP, and a decrease in the amount of the drug that was involved in metabolism. (3) The degree of inhibition of metabolic enzymes and transporters was greater than the effect of elevated free drug concentrations in the uremic patients and thus the metabolic process of atorvastatin was inhibited.

There is still disagreement regarding the relationship between total atorvastatin blood concentration and the occurrence of adverse reactions. A study has shown that an increased concentration of atorvastatin increases the risk of adverse reactions, such as rhabdomyolysis and liver damage (30). However, in a study among 61 lipid-controlling patients with detectable serum atorvastatin concentrations, 7 patients had myalgia and their serum concentrations were not significantly greater than those in patients without myalgia (31). However, this study did not indicate the baseline status and biochemical indicators of the enrolled patients. Consideration of variations in free drug concentrations and protein binding rates may lead to abnormal tissue distribution concentrations and may be one way to explain this discrepancy. Thus far, we have not found any research correlating the free drug concentration of atorvastatin with an incidence of adverse reactions. Since our study included uremic patients who were receiving regular hemodialysis treatment at dialysis centers, these patients typically take atorvastatin for a long time and have good tolerance, with no specific adverse reactions observed. Further multi-center, large-sample studies are still needed to confirm this correlation.

## Conclusion

Despite the fact that the metabolic process of atorvastatin may be inhibited in uremic hemodialysis patients, the total concentration of atorvastatin did not change significantly, but due to the decrease of protein binding rate, the free drug concentration may fluctuate drastically, and the increase in free drug concentration will increase the drug distribution in the liver or muscle tissue, which may increase the risk of certain adverse reactions. We recommend that clinicians use free drug concentration monitoring to adjust the dose of atorvastatin to ensure patient safety for uremic hemodialysis patients, especially for those with high-risk factors that may lead to serious adverse effects such as rhabdomyolysis or hepatic injury.

## Data Availability

The datasets presented in this study can be found in online repositories. The names of the repository/repositories and accession number(s) can be found in the article/Supplementary Material.
